# The prevalence and correlates of depression among older adults in greater Kumasi of the Ashanti region

**DOI:** 10.1186/s12889-023-15361-z

**Published:** 2023-04-25

**Authors:** Emmanuel K. Nakua, John Amissah, Phyllis Tawiah, Bernard Barnie, Peter Donkor, Charles Mock

**Affiliations:** 1 Department of Epidemiology and Biostatistics, Kwame Nkrumah University of Science and Technology, Kumasi, Ghana; 2 Department of Surgery, Kwame Nkrumah University of Science and Technology, Kumasi, Ghana; 3 Department of Medicine, Kwame Nkrumah University of Science and Technology, Kumasi, Ghana; 4 University of Washington, Seattle, USA

**Keywords:** Depression, Prevalence, Geriatric Depression Scale, Older adults, Ghana

## Abstract

**Background:**

Approximately two million Ghanaians suffer from mental disorders including depression. The WHO defines it as an illness characterized by constant sadness and loss of interest in activities that a person usually enjoys doing and this condition is the leading cause of mental disorders; however, the burden of depression on the aged population is fairly unknown. A better appreciation of depression and its predictors is necessary to design appropriate policy interventions. Therefore, this study aims to assess the prevalence and correlates of depression among older people in the Greater Kumasi of the Ashanti region.

**Methods:**

A cross-sectional study design with a multi-stage sampling approach was employed to recruit and collect data from 418 older adults aged 60 years and above at the household level in four enumeration areas (EAs) within the Asokore Mampong Municipality. Households within each EAs were mapped and listed by trained resident enumerators to create a sampling frame. Data was collected electronically with Open Data Kit application over 30 days through face-to-face interaction using the Geriatric Depression Scale (GDS). The results were summarized using descriptive and inferential statistics. A multivariable logistics regression using a forward and backward stepwise approach was employed to identify the predictors of depression in the study sample. All analyses were performed using STATA software version 16, and the significance level was maintained at a p-value < 0.05 and presented at a 95% confidence interval.

**Results:**

The study achieved a response rate of 97.7% from the estimated sample size of 428 respondents. The mean age was 69.9 (SD = *8.8)*, and the distribution was similar for both sexes (p = 0.25). The prevalence of depression in this study was 42.1% and dominated by females, older adults (> 80 years), and lower economic class respondents. The rate was 43.4% for both consumers of alcohol and smokers with a history of stroke (41.2%) and taking medication for chronic conditions (44.2%). The predictors of depression in our study were being single, low class [aOR = 1.97; 95% CI = 1.18–3.27] and having other chronic conditions [aOR = 1.86; 95% CI = 1.59–4.62], and the inability to manage ones’ own affairs [aOR = 0.56; 95% CI = 0.32–0.97].

**Conclusion:**

The study provides data that can inform policy decisions on the care of the elderly with depression in Ghana and other similar countries, confirming the need to provide support efforts towards high-risk groups such as single people, people with chronic health conditions, and lower-income people. Additionally, the evidence provided in this study could serve as baseline data for larger and longitudinal studies.

## Background

The life expectancy of countries continues to increase despite setbacks from global pandemics such as HIV and Covid-19 [1, 2], especially in Africa. The significant growth in the aging population could be attributed to improvements in ageing care and the general healthcare systems [3–5]. Global estimates suggest that there are 900 million persons aged 60 years and older currently and this figure is projected to double from 12% currently to 22% by the year 2050, translating to approximately 2 billion [4–6]. Likewise, the adult population above 60 years in Africa including Ghana is expected to increase from 48 million to 207 million by 2050 [4]. This means that 1-in-6 of the African population will be aged 65 years or over by the mid-century. The Ghanaian population could be described as relatively younger as 6.7% of the population is over 60 years, which translates to about 1.6 million people [7, 8]. Notwithstanding, a United Nations projection suggests that the Ghanaian population above 60 years will be between 9.7 − 14.1% of the population by the year 2050 [3, 4].

Aging is a natural phenomenon that presents with its challenges and is thus a public health concern. As individual advances in age, they begin to experience certain reduced functionalities [6, 9]. The presence of certain biological and neurological risk factors increases their susceptibility to both physical and mental health conditions including depression [10–14]. Depression is the leading cause of mental health-related morbidity and mortality globally including Ghana [6, 15–18]. While depression could affect anyone, it has been reported to be more prevalent in the older population affecting 7% of those aged 60 + globally [6, 14] and accounting for 5.7% of the individual’s years of living with disability (YLD) [6, 12].

The WHO defines depression as a mental condition that is characterized by a persistent state of sadness, and a loss of interest in a formally perceived rewarding or enjoyable activity and could affect an individual’s concentration, eating, and sleeping behavior [16, 19]. Depression negatively affects the individual’s social and physical functioning [14, 20–22] and usually presents with a loss of interest in activities, loneliness, mood swings, changes in one’s sleeping pattern, loss of sense of self-worth, and suicidal thoughts, especially in the much older adults [19, 23]. Estimates from the WHO report depression to be the major contributor to 800 000 deaths from suicide yearly [19].

The burden of depression in the older adult population has been variedly documented in developed countries like America (9.8-11.2%) [9, 24], England (8.7%) [25], Australia (8.2%) [26] and China (2.2-10.5%) [27–29]. Other estimates from the West suggest that up to 16% of its population shows depressive symptoms [30, 31]. Africa is not spared of this burden; for instance, in Ethiopia, the burden ranged between 28.5-45% [32–34]; 37.5-44.4% in Egypt [35, 36], 19-29.3% in Uganda [37, 38], 44.7% in Nigeria [39] and Sudan (47.5%) [40]. These rates are alarming and call for the need to identify all the factors that contribute to this rise in figures across the continent. Factors such as age [34, 41], sex [34, 38, 41–43], wealth status [13, 37, 44], poor health status [28], the existence of co-morbid conditions [12, 23, 45, 46] and modifiable lifestyle behaviors such as dietary intakes, alcohol consumption, smoking, living sedentary lifestyle [45, 47–50] have been consistently reported to be correlates of depression in the developed countries [36, 41].

Despite the overwhelming burden of depression reported elsewhere, data in Ghana is limited and the few existing ones remain scanty. A few comparative studies from the decade-old WHO’s Study on Global Ageing and Adult Health (SAGE Wave 1) data reported depression prevalence to be between 6.7 and 13.6% in the Ghanaian population [41, 46, 51].

The other existing studies did not report on the general population but focused on specific populations with conditions such as diabetes [45] and HIV [51]. Consistently reporting more on depression prevalence and identifying its associated factors will enable us to create a pool of evidence that will be of significance to policymakers, academics, and service providers. To the best of our knowledge, evidence on adults’ depression is still low compared to other health conditions which makes it impossible for policymakers to appreciate its burden in Ghana. Inadequate evidence may undermine the urgency to prioritize depression in the mental health service at the primary level of our healthcare system. This study aimed to assess the prevalence and correlates of depression among older adult in the Greater Kumasi of the Ashanti region using the Geriatric depression scale. By so doing, we hoped to add to the currently limited body of evidence on the extent of geriatric depression in Africa using a housing-a-household survey design.

## Methodology

A cross-sectional study design was employed to collect data from older adults at the household level in the Asokore Mampong Municipality from 1st − 30th June 2021. The Asokore Mampong Municipality was selected due to its heterogeneous population, as parts of it are rural, peri-urban, and urban, which is a representation of the characteristics of the Ashanti region.

The study recruited both men and women aged 60 years and above who have been residing in the Municipality for at least the last 12 months prior to the survey. A sample size of 428 individuals was estimated using the Cochrane sample size estimation formulae [52]. However, this analysis included 418 individuals, given a response rate of 97.7%. Evidence from earlier studies indicates that 5.7% of older adults above 60 years are more susceptible to experiencing depression than those below that threshold [19].

The PMA2020 sampling frame was used for this study. A multi-stage sampling approach was employed to recruit participants for the study. The PMA2020 sampling methodology was adopted, and 4 enumeration areas (EA) located within the Asokore Mampong Municipality were selected from the original 100 enumeration areas used for the national survey. Details of the PMA2020 Ghana sampling approach on how the 100 enumeration areas were created is published elsewhere [53]. Firstly, the households within each of the selected EAs in the Asokore Mampong were mapped and listed by eight trained resident enumerators (RE) to create a sampling frame. Each EA had approximately 200 households from which the participants were selected. Secondly, a random selection of the listed households with the aid of the “Random Number Generator” mobile phone application was used to select households in each EA. Two resident enumerators were assigned to an EA in the municipality for the data collection exercise. Households with eligible participants were recruited for the study. The REs visited the selected households and introduced the study and those who consented were interviewed. In instances where there was more than one eligible household member, they were all interviewed. In situations where the eligible participant was not available, another interview appointment was scheduled and on a third visit, if they are still absent, that household was skipped. No replacement was done where eligible respondents in a household could not be interviewed.

### Data collection

Data was collected over 30 days through face-to-face interaction using a self-drafted structured questionnaire, instrumental activities of daily living scale (IADLS), and the Geriatric Depression Scale (GDS). The tools were programmed into an electronic format and loaded onto a smartphone using the Open Data Kit (ODK) application [54, 55]. A 3-day intensive training was organized for the REs on mobile data collection, the content of the tools, and how to maintain covid-19 protocols during the data collection. The training involved the translation of the questionnaire into the local dialect to maintain consistency with the meaning after which it was pre-tested in a different municipality (Oforikrom). The questions that came out unclear were identified and addressed before the actual data collection exercise. The interviews were conducted in a private environment devoid of distractions in the participant’s preferred language either in English or the local dialect mainly “Twi”.

### Data collection tool

The 8-item IADLs is a validated tool that has been used in successfully several community-based studies in different settings in sub-Saharan Africa among the aged population including Ghana [13, 56]. Additionally, the GDS is a 15-item scale that has been used widely to assess depression in the aged population [33, 57]. This scale was specifically designed for interviewing the older population not based on any physical symptoms of depression [58–60]. The GDS is easy to administer in different settings including the household levels to assess depression. It has been extensively tested in low-middle-income countries including Ethiopia, India, Europe and other Asian countries [23, 33, 61, 62] and it is reported to have a sensitivity and specificity of above 92% and 89% respectively in the Asian and European adult populations [61, 62]. The GDS has not been validated in any Ghanaian Setting to the best of our knowledge, nevertheless study employed rigorous measures including experts engagement to translate the questionnaire into the local language with aid of TWI Medical Glossary for clinicians and health workers developed by the Kwame Nkrumah University of Science and Technology as a guide [63].

The sum score for this scale is 15 points and each question has a binary response of *‘Yes’ or ‘No’.* Ten items in the scale were positively worded such that a response of Yes is assigned a value of 1 and a ‘No’ is assigned ‘0’. On the other hand, the remaining five items were negatively worded, so the “No” response had a value of 1 and vice versa. The depression scale is categorized based on the cut-off scores which are “Non-depressed” (1–5 scores) and “Depressed” (6–15 scores) [34, 57]. Other information includes the participant’s socio-economic activities, lifestyle activities such as smoking and consumption of alcohol, health status including the presence of hypertension, stroke, and diabetes, the presence of support systems such as caretaker availability, and their ability to perform some of the instrumental activities of daily living (IADLS).

### Data analysis

Data were summarized using both descriptive and inferential statistics. Continuous variables were summarized with means and standard deviation while categorical variables were presented as frequencies and percentages. A wealth index of the household was constructed based on the ownership of five basic assets/items (Television, refrigerator, fan, radio/stereo, mobile phone), availability of toilet facility, conditions on the structure they lived in (material used for the floor and wall of the structure). This index was constructed using principal component analysis and was categorized into three classes (High, middle, lower). Depression was operationalized as the presence of variables/symptoms that contribute to a score above the threshold considered normal. The dependent variable was the presence of depression (GDS ≥ 6) irrespective of the category. A chi-square (*X*^2^) was used to assess the sex differences in the socio-demographic and other risk factors. A multivariable logistics regression using a forward and backward stepwise approach was employed to identify factors that predicted possible depressions in the study sample. All analyses were performed using STATA software version 16 and the significance level was maintained at p-value < 0.05 and presented at a 95% confidence interval.

## Results

### Characteristics of the study sample

The mean age of the study sample was 69.9 years *± 8.8* and the distribution was similar for both sexes (p = 0.25). Being single was remarkably higher among the female participants than their male counterparts (p = 0.001). Educational status varies significantly between both sexes; 3-in-4 of the females had no formal education (76.5%). There was a difference in the religious affiliation and employment status of the men and women in this study, however, the wealth index significantly varied among the two groups (p = 0.001). Risky lifestyle behavior such as alcohol consumption and smoking varied among the two sexes; the male group dominated in both activities (p = 0.001). The number of women with co-morbidities such as diabetes (p = 0.004), hypertension (p = 0.001), and other chronic conditions (p = 0.046) was high compared to their men counterparts. The prevalence of stroke was high among men compared to women; however, the difference was not significant (p = 0.176). Other factors such as taking routine medication for these chronic conditions, the availability of a caregiver/caretaker, and the number of hours a caretaker spent with the participants were similar for both men and women. Other instrumental activities of daily living such as managing traveling and transportation and finance did not differ significantly between the two sexes (Table 1).

**Table 1:** Demographic characteristics of the study sample by sex difference

Variables	(%) N = 418	Male (%) *n = 134*	Female (%) *n = 284*	${\varvec{x}}^{2}$	p-Value
**Age group**				2.75	0.25
60–70	260 (62.2)	79 (59.0)	181 (63.7)		
71–80	101 (24.2)	39 (29.1)	62 (21.8)		
Above 81	57 (13.6)	16 (11.9)	41 (14.4)		
Mean age *(SD)*	*69.9 ± 8.8*				
**Marital status**				68.87	0.001
**¥** Married	189 (45.2)	100 (74.6)	89 (31.3)		
**#** Single	229 (54.8)	34 (25.4)	195 (68.7)		
**Educational status**					
Formal education	192 (45.9)	81 (60.4)	111 (39.1)	16.73	0.001
No formal education	226 (54.1)	53 (39.6)	173 (60.9)		
**Religion**					
Christianity	167 (39.9)	56 (41.8)	111 (39.1)	1.64	0.44
Islam	243 (58.1)	74 (55.2)	169 (59.5)		
Traditional religion	8 (1.9)	4 (2.9)	4 (1.4)		
**Current Employment status**				0.12	0.72
Employed	117 (27.9)	39 (29.1)	78 (27.5)		
Not employed	301 (72.1)	95 (70.9)	206 (72.5)		
**Wealth Tercile**					
Lower class	149 (36.1)	32 (24.8)	117 (41.2)	12.55	0.002
Middle class	135 (32.7)	44 (34.1)	91 (32.0)		
High class	129 (31.2)	53 (41.1)	76 (26.8)		
**Ever Smoke**				27.51	0.001
No	362 (86.6)	99 (73.9)	263 (92.6)		
Yes	56 (13.4)	35 (26.1)	21 (7.4)		
**Consume Alcohol**				31.46	0.001
No	358 (85.7)	96 (71.6)	262 (92.2)		
Yes	60 (14.4)	38 (28.4)	22 (7.8)		
**Hypertension**				26.55	0.001
No	201 (48.1)	89 (66.4)	112 (39.4)		
Yes	217 (51.9)	45 (33.6)	172 (60.6)		
**Diabetes**				8.09	0.004
No	353 (84.5)	123 (91.8)	230 (80.9)		
Yes	65 (15.5)	11 (8.2)	54 (19.1)		
**Ever suffer stroke**				1.83	0.176
No	401 (95.9)	126 (94.0)	272 (96.3)		
Yes	17 (4.1)	8 (6.0)	9 (3.7)		
**Other Chronic condition**				3.99	0.046
No	360 (86.1)	122 (91.0)	238 (83.8)		
Yes	58 (13.9)	12 (9.0)	46 (16.2)		
**Taking of medication for chronic conditions**					
No	58 (22.8)	18 (30.0)	40 (20.5)	2.35	0.125
Yes	197 (77.2)	42 (70.0)	155 (79.5)		
**Caretaker available**				2.66	0.10
No	306 (73.2)	105 (78.4)	201 (70.8)		
Yes	112 (26.8)	29 (21.6)	83 (29.2)		
**Number of Hours spent with caretaker**				0.17	0.91
1–6 h	17 (15.2)	5 (17.2)	12 (14.5)		
7–12 h	30 (26.8)	8 (27.6)	22 (26.5)		
13 + hours	65 (58.0)	16 (55.2)	49 (59.0)		
**Ability to manage traveling and transportation by self**				8.42	0.006
No	67 (16.0)	15 (11.2)	52 (18.3)		
Yes	351 (84.0)	119 (88.8)	232 (81.7)		
**Ability to manage finance by self**				27.51	0.012
No	23 (5.5)	4 (3.0)	19 (6.7)		
Yes	395 (94.5	130 (97.0)	265 (93.3)		

**¥ Married**: cohabiting, married formal/informal

**# Single**: Never married, divorced, separated, widowed

### Socio-demographic-specific prevalence rates of depression

The prevalence of depression rates estimated for the various socio-demographic factors and independent variables are presented in Table 1. Per the case definitions and cut-off points from the GDS, 172/418 (42.1%) respondents could be identified as “depressed cases”. The prevalence rate was higher in the female participant than in males (46.1% versus 33.6%) and higher among the age group 81 years and above (61.4%). The rate was high among participants with no education (44.7%); single (49.8%); Muslims (41.6%); unemployed (43.5%) and participants in the lower class (51.7%) and the middle class (43.0%).

### Other independent variables prevalence rates of depression

The prevalence rate of depression was estimated among participants with certain risk factors versus those without these factors (Table 1). The rate was low among smokers (43.4%); participants who have ever suffered a stroke (41.2%); those taking medications for their chronic conditions (44.2%), those with caretakers/caregivers available with them (38.4%); those with the ability to manage their traveling and transportation (39.3%), and finance (40.5%) compared to their counterparts. Consumers of alcohol (43.3%), participants who have hypertension (47.0%), diabetes (46.2%), other chronic conditions (56.9%), and have their caregiver/care takers spend long hours with them beyond 13 h (44.6%) showed higher depression rates compared to their counterparts.

### Predictors of depression

In the univariable analysis, educational status and being diabetic predicted the risk of depression. From the multivariate analysis, variables such as marital status, wealth tercile, other chronic conditions, the ability of participants to manage their travels and transportation, and finance without any aid were statistically significant predictors of depression among our study sample. (Table 2).

**Table 2:** Factors predicting depression among senior citizens in Greater Kumasi of the Ashanti Region

Variables					Univariate Model				Multivariate Model		
	Total	Non-Depressed	Depressed		Crude Odd Ratio (OR; 95% CI)		p-Value		Adjusted Odd Ratio (aOR; 95% CI)		p-Value
**Age group**											
60–70	260 (62.2)	152(58.5)	108(41.5)		1						
71–80	101 (24.2)	62(61.4)	39(38.6)		0.82 [0.50–1.32]		0.42		-		
Above 81	57 (13.6)	28(49.1)	29(50.9)		1.40 [0.78–2.49]		0.24		-		
**Sex**											
Male	134 (32.1)	89(66.4)	45(33.6)								
Female	284(67.9)	153(53.9)	131(46.1)								
**Marital status**											
**¥** Married	189(45.2)	127(67.2)	62(32.8)		1						
**#** Single	229(54.8)	115(50.2)	114(49.8)		1.91 [1.28–2.86]		0.002		1.66 [1.08–2.53]		**0.018**
**Educational status**											
Educated	192(45.9)	117(60.9)	75(39.1)		1						
No education	226(54.1)	125(55.3)	101(44.7)		0.77[0.52–1.14]		0.20		0.68 [0.36–1.30]		0.25
**Religion**											
Christianity	167(39.9)	96(57.5)	71(42.5)		1						
Muslim	243(58.1)	142(58.4)	101(41.6)		1.35[0.32–5.61]		0.67		-		
Traditional religion	8 (1.9)	4(50.0)	4(50.0)		0.99[0.66–1.48]		0.98		-		
**Current Employment status**											
Employed	117(27.9)	72(61.5)	45(38.5)		1						
Not employed	301(72.1)	170(56.5)	131(43.5)		0.81[0.52–1.26]		0.37		-		
**Wealth Tercile**											
Lower class	149(36.1)	72(48.3)	77(51.7)		2.15 [1.32–3.51]		0.002		1.97[1.18–3.27]		**0.009**
Middle class	135(32.7)	77(57.0)	58(43.0)		1.44 [0.87–2.38]		0.15		1.37[0.82–2.30]		0.22
High class	129(31.2)	89(69.0)	40 (31.0)		1		-				
**Ever Smoke**											
No	362(86.6)	205(56.6)	157(43.4)		1						
Yes	56(13.4)	37(66.1)	19(33.9)		0.61 [0.33–1.12]		0.11		-		
**Consume Alcohol**											
No	358(85.7)	208(58.1)	150(41.9)		1						
Yes	60(14.4)	34(56.7)	26(43.3)		1.47[0.48–4.46]		0.49		-		
**Hypertension**											
No	201(48.1)	127(63.2)	74(36.8)		1						
Yes	217(51.9)	115(53.0)	102(47.0)		1.52 [1.02–2.27]		0.03		1.45[0.96–2.20]		0.04
**Diabetes**											
No	353(84.5)	207(58.6)	146(41.4)								
Yes	65(15.5)	35(53.8)	30(46.2)		1.18 [0.69–2.03]		0.04		1.18[0.64–2.18]		0.58
**Ever suffer stroke**											
No	401(95.9)	232 (57.9)	169(42.1)								
Yes	17(4.1)	10(58.8)	7 (41.2)								
**Other Chronic condition**											
No	360(86.1)	217(60.3)	143(39.7)		1						
Yes	58(13.9)	25(43.1)	33(56.9)		1.91 [1.07–3.39]		0.02		1.86[1.59–4.62]		0.04
**Taking of medication for chronic conditions**											
No	58(22.8)	27(46.6)	31(53.4)		1						
Yes	197(77.2)	110(55.8)	87(44.2)		0.65 [0.35–1.19]		0.17		-		
**Caretaker available**											
No	306(73.2)	173(56.5)	133(43.4)		1						
Yes	112(26.8)	69(61.6)	43(38.4)		0.80 [0.51–1.26]		0.34		-		
**Number of Hours spent with caretaker**											
1–6 h	17(15.2)	11(64.7)	6 (35.3)		1						
7–12 h	30(26.8)	22(73.3)	8 (26.7)		0.69 [0.19–2.52]		0.58		-		
13 + hours	65(58.0)	36(55.4)	29(44.6)		1.47 [0.48–4.47]		0.49		-		
**Ability to manage traveling and transportation by self**											
No	67(16.0)	29(43.3)	38(56.7)		1						
Yes	351(84.0)	213 (60.7)	138(39.3)		0.51 [0.30–0.88]		0.01		0.56[0.32–0.97]		**0.041**
**Ability to manage finance by self**											
No	23(5.5)	7(30.4)	16(69.6)		1						
Yes	395(95.5)	235(59.5)	160(40.5)		0.30 [0.12–0.75]		0.01		0.33[0.11–0.97]		**0.04**

**¥ Married**: cohabiting, married formal/informal

**# Single**: Never married, divorced, separated, widowed

Single participants were [aOR = 1.66; 95% CI = 1.08–2.53] more likely to be depressed compared to their married counterparts. The likelihood of depression decreased as the participant’s economic status increased. Those in the lower class of the wealth tercile were 1.97 times [aOR = 1.97; 95% CI = 1.18–3.27] more likely to experience depression compared to those in the upper class. Also, participants with other chronic conditions were [aOR = 1.86; 95% CI = 0.59–4.62] more likely to be reported as depressed. Two instrumental activities of daily living (IADL) were identified to influence the possibility of depression among the participants. The participant’s ability to manage their traveling and transportation arrangement reduces their chances of being reported as depressed by 44% [aOR = 0.56; 95% CI = 0.32–0.97] compared to those who need assistance to perform that task. Similarly, the ability of participants to manage their finances reduces the likelihood of being depressed by 67% [aOR = 0.33; 95% CI = 0.11–0.97].

## Discussion

Increased longevity in the geriatric population is desired in every society, however, ageing comes with its challenges. This stage encompasses a rise in the burden of chronic non-communicable diseases including depression and any attempt to design a support system required the need to understand the burden of depression among older adults. The study found that 2-in-5 of the study sample had some form of depression. Depression was more prevalent in females, of older age (> 81 years), those with no formal education, Muslims, unemployed, single participants, and those within the middle class. The major predictors of depression on multivariable analysis were being single, low class in the wealth tercile, having other chronic conditions, and the inability to manage own affairs (arranging travels, transportation, and finances by self).

The prevalence of depression found in this study was 42.1%. This rate is consistent with the rates reported from other community-based studies conducted in Nigeria (44.7%), Egypt (44.4%), Ethiopia (41.8%), and Sudan (47.5% [32–34, 36, 38–40, 63]. However, the finding was 4 times higher compared to an earlier rate reported in Ghana, where depression was estimated to be between 6.7 and 13.6% [41, 46, 51]. This variation could be explained by the difference in the operational definitions of depression in both cases and the population dynamics. Our study used the Geriatric Depression Scale whereas the earlier studies conducted in Ghana used the symptoms-based questionnaire and these tools have their unique thresholds for classifying depressed and non-depressed cases [41, 46, 51]. Undoubtedly the Ghanaian population over the past decade has seen significant changes including its size and social structure, and these changes could reflect in their physical, psychological, and mental well-being. Therefore, it is no surprise to see a change in the depression rate after a decade of population dynamics.

Consistently depression was more prevalent among females (46.1%) participants and those advanced in age (81 years and above) similar to studies conducted elsewhere [34, 38, 41–43]. For instance, Mulat et al., 2021 [34] and Ofori-Atta et al., 2011 [42] in separate studies reported that women were more likely to become depressed than men and this could be attributed to the inherent vulnerability and gender disadvantage among female population. This disparity could relate to how inequality explains depression among women. The burden of depression was among the octogenarians in this study, and this finding corroborates with evidence from early literature [34, 41]. Old age is usually associated with reduced functionality and other chronic conditions and these health conditions lead to some disabilities which are known to contribute to about 30% of the psychiatric conditions among the aged [6, 15, 17, 18, 64, 65]. Also, the presence of economic loss, dependency on caretakers, and perception about one’s self-worth associated with older age sometimes make life unpleasant for the individual in question which eventually affects their psychological well-being [11]. Even though it is evident that the social support system is protective against developing depression, such support may be well appreciated if it comes from a professional. Most caretakers in the Ghanaian cultural settings are usually young family members who may have to leave their jobs to spend time with the aged, a situation that could heighten the feeling of helplessness in the aged. It is evident that caregivers who spend long hours with the respondents may also experience psychological distress and burnout after a while and tend to abuse their victims, a situation that could heighten the depressive states of those they care for [10] This could explain why our study found a high depression rate among people who spent more hours with a caretaker.

Additionally, the burden of depression was higher among single participants and this finding is consistent with similar studies conducted in India [23], Ethiopia [33], and Uganda [37]. Specifically, single participants were 1.66 times more likely to record depression compared to those in relationships either married or cohabiting. Being married or co-habiting serves as one of the surest ways older adults could provide social support in the form of companionship to each other, whereas single people may lack such support and eventually suffer what Amegbor et al., 2020 in their study termed as social isolation. To Amegbor et al., 2020, this support system protects older adults from the onset of social isolation, loneliness, and eventually depression [13].

The burden of depression was higher among participants with socio-demographic factors such as belonging to the lower class in the community and these findings corroborate with an earlier study in Ghana which reported similar findings [46].

The factors predicting depression in our study included socio-demographic characteristics (marital status and wealth tercile), morbidity (other chronic conditions), and ability to manage one’s finances; manage own traveling and transportation without aid.

Wealth status was protective against depression in our study; the likelihood of being depressed was reduced among participants in higher wealth class and vice versa and this finding is consistent with other studies conducted elsewhere [13, 23, 46, 51]. Our study does not entirely suggest that people in the high-wealth class cannot be depressed, rather their risk is reduced compared to those in the lower class. Generally, the socio-economic status of individuals determines their purchasing power and eventually access to healthcare including mental health support before they develop any problem.

Participants with known other chronic conditions were more likely to suffer from depression compared to their counterparts. This finding is consistent with evidence reported in the literature [12, 23, 45, 46]. The presence of co-morbid conditions in the adult population makes them prone to depression. For instance, a WHO report suggested that the presence of co-morbidities increases an individual’s susceptibility to disabilities which are known to be a major predictor of psychological disorders [9]. Co-morbid conditions such as hypertension, diabetes, and stroke in an older adult usually limits their ability, and increase their disability and dependency on others. The feeling of helplessness in these populations causes them to develop psychological conditions including depression. Also, other chronic conditions such as asthma, arthritis, and backache in the current study were found to have an association with developing depression. Participants with other chronic conditions were almost twice likely to be identified as depressed compared with the latter, this is consistent with another study report from India [23].

In our present study, we found out that, the ability of an individual to manage their own travels and transportation, and financial transactions daily without assistance were protective against being depressed. Participants with the ability to arrange their transportation and travel had a 44% reduced chance of developing depression compared to those who need support. Likewise, those able to handle their finances without help have 67% protection from being depressed. The ability of the older adult to handle some of the basic activities of daily living is an indication of having their cognition intact. It also gives them some sense of hope and belief that they are able, and their lives are still normal. Consistently, evidence from aging reports suggests that reduced functioning among the adult population increases their susceptibility to developing a psychological disorder including depression, and vice versa [6, 11–13, 23]. For instance, Chauhan et al., 2016 [23] reported in their study that adults with low scores in the activities of daily living were more depressed than the able ones. Similarly, Mirkena et al., 2018 argued in their study conducted in Ethiopia that adults who were dependent on their children to carry out basic daily activities were three-fold more likely to be depressed compared to the able ones [33].

### Limitations of the study

The study was limited in scope, target population, and possible recall bias. The study was limited to the aged population residing in one district without involving the entire region. Additionally, only community dwellers were included leaving out older adults living in institutional homes; a situation that could cause us to miss other depressed victims. Questions assessing wealth terciles, medical history, smoking behavior, and alcohol consumption are considered sensitive issues to discuss in traditional settings, and responses could be subjected to a bias. Nevertheless, adequate scientific scrutiny such as the randomization technique employed, proper community entry, building a good rapport, ensuring confidentiality and privacy during data collection rigorous statistical analysis were employed to reduce these limitations.

## Conclusion

Our study revealed that geriatric depression was high which is a public health concern. Marital status, wealth status, the presence of known co-morbid, and other chronic conditions were determining factors for the onset of depression. Also, the ability to perform ones’ basic activities of daily living such as managing own travels, arranging transportation and finance without any aid was protective against developing depression. The study provides data that can inform policy decisions on the care of older adults with depression in Ghana and other similar countries, such as confirming the need to provide support efforts towards high-risk groups such as single people, people with chronic health conditions, and lower-income people. Additionally, the evidence provided in this study could serve as baseline data for larger and longitudinal studies.

## Data Availability

The dataset used for this study is in the custody of the corresponding author and will be made available upon a reasonable request.

## References

[1] Parker A, Koegelenberg CFN, Moolla MS, Louw EH, Mowlana A, Nortjé A et al. High HIV prevalence in an early cohort of hospital admissions with COVID-19 in Cape Town, South Africa. SAMJ: South African Medical Journal. 2020 Oct 1;110(9):0–0. 10.7196/SAMJ.2020.v110i10.15067 33205724

[2] Lukman AF, Rauf RI, Abiodun O, Oludoun O, Ayinde K, Ogundokun RO. COVID-19 prevalence estimation: four most affected african countries. Infect Dis Model. 2020 Jan;1:5:827–38. 10.1016/j.idm.2020.10.002 PMC7550075 33073068

[3] United Nations, World Population Ageing 2019 (2019). World Population Ageing 2019.

[4] United Nations:Department of Economic and Social affairs (DESA). World Population Ageing 2020: Highlights [Internet]. World Population Ageing 2020: Highlights. 2021 [cited 2021 Sep 7]. Available from: https://www.un.org/development/desa/pd/

[5] World Health Organization-WHO. Active aeging: a policy framework. World Health Organization; 2002.

[6] World Health Organinization (WHO). Mental health of older adults [Internet]. [cited 2021 Sep 9]. Available from: https://www.who.int/en/news-room/fact-sheets/detail/mental-health-of-older-adults

[7] Ghana Statistical Service. 2010 Population and Housing Census, District Analytical Report- Kumasi Metropolis.Ghana Statistial Service. 2014

[8] Ghana Statistical Services. Ghana Labour Statistics: Economic activity status of persons 15 years and older by region. Labour Statistics. 2010.

[9] Mohebbi M, Agustini B, Woods RL, McNeil JJ, Nelson MR, Shah RC et al. Prevalence of depressive symptoms and its associated factors among healthy community-dwelling older adults living in Australia and the United States. Int J Geriatr Psychiatry [Internet]. 2019 Aug 1 [cited 2021 Sep 11];34(8):1208–16. Available from: https://onlinelibrary.wiley.com/doi/full/10.1002/gps.5119 10.1002/gps.5119 PMC6924573 30989707

[10] Orfila F, Coma-Solé M, Cabanas M, Cegri-Lombardo F, Moleras-Serra A, Pujol-Ribera E. Family caregiver mistreatment of the elderly: Prevalence of risk and associated factors.BMC Public Health. 2018 Jan22;18(1):1–14. 10.1186/s12889-018-5067-8 PMC5778739 29357866

[11] Fiske A, Wetherell JL, Gatz M. Depression in Older Adults. http://dx.doi.org/101146/annurev.clinpsy032408153621 [Internet]. 2009 Mar 27 [cited 2021 Sep 9];5:363–89. Available from: https://www.annualreviews.org/doi/abs/10.1146/annurev.clinpsy.032408.153621 10.1146/annurev.clinpsy.032408.153621 PMC2852580 19327033

[12] Alexopoulos GS. Depression in the elderly.The Lancet. 2005 Jun4;365(9475):1961–70. 10.1016/S0140-6736(05)66665-2 15936426

[13] Amegbor PM, Kuuire VZ, Yawson AE, Rosenberg MW, Sabel CE. Social Frailty and Depression Among Older Adults in Ghana: Insights from the WHO SAGE Surveys: https://doi.org/101177/0164027520946447 [Internet]. 2020 Aug 4 [cited 2021 Sep 12];43(2):85–95. Available from: https://journals.sagepub.com/doi/abs/10.1177/0164027520946447?journalCode=roaa&journalCode=roaa 10.1177/0164027520946447 32748698

[14] Peltzer K, Phaswana-Mafuya N. Depression and associated factors in older adults in South Africa.Glob Health Action.2013;6(1). 10.3402/gha.v6i0.18871 PMC3549465 23336621

[15] Mogga S, Prince M, Alem A, Kebede D, Stewart R, Glozier N et al. Outcome of major depression in Ethiopia: Population-based study. The British Journal of Psychiatry [Internet]. 2006 Sep [cited 2021 Sep 9];189(3):241–6. Available from: https://www.cambridge.org/core/journals/the-british-journal-of-psychiatry/article/outcome-of-major-depression-in-ethiopia/F9FA142714D8584D3B896EFA2D9553A3 10.1192/bjp.bp.105.013417 16946359

[16] Patel V, Abas M, Broadhead J, Todd C, Reeler A. Depression in developing countries: lessons from Zimbabwe. BMJ. 2001 Feb;24(7284):482–4. 10.1136/bmj.322.7284.482 PMC1119689 11222428

[17] Dunn LW, Adebowale TO, Umapthy C, Chaudry IB, Yangye SD, Rahimi YA, et al. Mental disorders in the developing world. BMJ. 1994 Jun;25(6945):1716.

[18] Abas M, Broadhead J. Mental disorders in the developing world. BMJ [Internet]. 1994 Apr 23 [cited 2021 Sep 9];308(6936):1052–3. Available from: https://www.bmj.com/content/308/6936/1052 10.1136/bmj.308.6936.1052 PMC2539966 8173419

[19] World Health Organization. Depression and Other Common Mental Disorders [Internet]. 2017 [cited 2022 Dec 11]. p. 24. Available from: https://www.who.int/publications/i/item/depression-global-health-estimates

[20] Tol WA, Komproe IH, Thapa SB, Jordans MJD, Sharma B, De Jong JTVM. Disability associated with psychiatric symptoms among torture survivors in rural Nepal. J Nerv Mental Disease. 2007 Jun;195(6):463–9. 10.1097/NMD.0b013e31802f5dac 17568293

[21] Thapa SB, Hauff E. Perceived needs, self-reported health and disability among displaced persons during an armed conflict in Nepal. Soc Psychiatry Psychiatr Epidemiol. 2012 Apr;47(4):589. 10.1007/s00127-011-0359-7 PMC3304067 21476014

[22] Bolton P, Neugebauer R, Ndogoni L. Prevalence of depression in rural Rwanda based on symptom and functional criteria. Journal of Nervous and Mental Disease. 2002 Sep 1;190(9):631–7. 10.1097/00005053-200209000-00009 12357098

[23] Chauhan P, Kokiwar P, Shridevi K, Katkuri S. A study on prevalence and correlates of depression among elderly population of rural South India. Int J Community Med Public Health [Internet]. 2016 [cited 2021 Sep 9];3(1):236–9. Available from: http://www.ijcmph.com

[24] Steffens DC, Fisher GG, Langa KM, Potter GG, Plassman BL. Prevalence of depression among older Americans: the Aging, Demographics and Memory Study. Int Psychogeriatr [Internet]. 2009 Oct [cited 2021 Sep 9];21(5):879–88. Available from: https://www.cambridge.org/core/journals/international-psychogeriatrics/article/abs/prevalence-of-depression-among-older-americans-the-aging-demographics-and-memory-study/EE4B2F1D48735F4083EABB716500DD71 10.1017/S1041610209990044 PMC2747379 19519984

[25] McDOUGALL FA, KVAAL K, MATTHEWS FE, JONES PAYKELE, DEWEY PB (2007). Prevalence of depression in older people in England and Wales: the MRC CFA Study. Psychol Med.

[26] Pirkis J, Pfaff J, Williamson M, Tyson O, Stocks N, Goldney R et al. The community prevalence of depression in older Australians.J Affect Disord. 2009May 1;115(1–2):54–61. 10.1016/j.jad.2008.08.014 18817976

[27] Chen R, Hu Z, Qin X, Xu X, Copeland JRM. A community-based study of depression in older people in Hefei, China—the GMS-AGECAT prevalence, case validation and socio-economic correlates. Int J Geriatr Psychiatry. 2004 May 1;19(5):407–13. 10.1002/gps.1103 15156541

[28] Chen R, Wei L, Hu Z, Qin X, Copeland JRM, Hemingway H. Depression in older people in rural China. Arch Intern Med [Internet]. 2005 Sep 26 [cited 2021 Sep 9];165(17):2019–25. Available from: https://jamanetwork.com/journals/jamainternalmedicine/fullarticle/486712 10.1001/archinte.165.17.2019 16186473

[29] Cong L, Dou P, Chen D, Cai L. Depression and Associated Factors in the Elderly Cadres in Fuzhou, China: A Community-based Study. Int J Gerontol. 2015 Mar 1;9(1):29–33.

[30] Blazer DG. Depression in the elderly: myths and misconceptions. Psychiatr Clin North Am. 1997 Mar;20(1):111–9. 10.1016/s0193-953x(05)70396-8 9139285

[31] Cole MG, Bellavance F, Mansour A. Prognosis of depression in elderly community and primary care populations: a systematic review and meta-analysis. Am J Psychiatry. 1999 Aug;156(8):1182–9. 10.1176/ajp.156.8.1182 10450258

[32] Girma M, Ebrahim J. Geriatric Depression in Ethiopia: Prevalence and Associated Factors. J Psychiatry [Internet]. 2016 [cited 2021 Sep 10];20(1):400. Available from: https://www.researchgate.net/publication/313316801

[33] Mirkena Y, Reta MM, Haile K, Nassir Z, Sisay MM. Prevalence of depression and associated factors among older adults at ambo town, Oromia region, Ethiopia. BMC Psychiatry 2018 18:1 [Internet]. 2018 Oct 18 [cited 2021 Sep 10];18(1):1–7. Available from: https://link.springer.com/articles/10.1186/s12888-018-1911-8 10.1186/s12888-018-1911-8 PMC6194620 30336773

[34] Mulat N, Gutema H, Wassie GT. Prevalence of depression and associated factors among elderly people in Womberma District, north-west, Ethiopia. BMC Psychiatry 2021 21:1 [Internet]. 2021 Mar 8 [cited 2021 Sep 10];21(1):1–9. Available from: https://bmcpsychiatry.biomedcentral.com/articles/10.1186/s12888-021-03145-x 10.1186/s12888-021-03145-x PMC7938572 33685419

[35] Ahmed D, el Shair IH, Taher E, Zyada F. Prevalence and predictors of depression and anxiety among the elderly population living in geriatric homes in Cairo, Egypt. J Egypt Public Health Assoc. 2014 Dec;89(3):127–35. 10.1097/01.EPX.0000455729.66131.49 25534177

[36] Afifi M. Depression in adolescents: Gender differences in Oman and Egypt [Internet]. Vol. 12, Eastern Mediterranean Health Journal. 2006 [cited 2021 Sep 12]. p. 61–71. Available from: https://pubmed.ncbi.nlm.nih.gov/17037222/17037222

[37] Kinyanda E, Woodburn P, Tugumisirize J, Kagugube J, Ndyanabangi S, Patel V. Poverty, life events and the risk for depression in Uganda. Social Psychiatry and Psychiatric Epidemiology 2009 46:1 [Internet]. 2009 Nov 16 [cited 2021 Sep 11];46(1):35–44. Available from: https://link.springer.com/article/10.1007/s00127-009-0164-8 10.1007/s00127-009-0164-8 PMC3432478 19916062

[38] Adewuya AO, Ola BA, Aloba OO, Mapayi BM, Oginni OO. Depression amongst Nigerian university students. Social Psychiatry and Psychiatric Epidemiology 2006 41:8 [Internet]. 2006 May 5 [cited 2021 Sep 11];41(8):674–8. Available from: https://link.springer.com/article/10.1007/s00127-006-0068-9 10.1007/s00127-006-0068-9 16680408

[39] Awunor NS, Ntaji MI, Edafiadhe EW, Erhabor PO, Eferakorho AO, Ijirigho B et al. Prevalence and predictors of depression among the elderly in selected rural communities in Delta State, Nigeria. Journal of Community Medicine and Primary Health Care [Internet]. 2018 Mar 23 [cited 2021 Sep 10];30(1):122–30. Available from: https://www.ajol.info/index.php/jcmphc/article/view/168672

[40] Assil SM, Zeidan ZA (2013). Prevalence of depression and associated factors among elderly sudanese: a household survey in Khartoum State. East Mediterr Health J.

[41] Thapa SB, Hauff E. Gender differences in factors associated with psychological distress among immigrants from low- and middle-income countries. Social Psychiatry and Psychiatric Epidemiology 2005 40:1 [Internet]. 2005 Jan [cited 2021 Sep 11];40(1):78–84. Available from: https://link.springer.com/article/10.1007/s00127-005-0855-8 10.1007/s00127-005-0855-8 15624079

[42] Ofori-Atta A, Cooper S, Akpalu B, Osei A, Doku V, Lund C et al. Common understandings of women’s mental illness in Ghana: Results from a qualitative study. http://dx.doi.org/103109/095402612010536150 [Internet]. 2011 Dec [cited 2021 Sep 11];22(6):589–98. Available from: https://www.tandfonline.com/doi/abs/10.3109/09540261.2010.536150 10.3109/09540261.2010.536150 21226647

[43] Ovuga E, Boardman J, Wasserman D. The prevalence of depression in two districts of Uganda. Social Psychiatry and Psychiatric Epidemiology 2005 40:6 [Internet]. 2005 Jun [cited 2021 Sep 11];40(6):439–45. Available from: https://link.springer.com/article/10.1007/s00127-005-0915-0 10.1007/s00127-005-0915-0 16003593

[44] Ettman CK, Abdalla SM, Cohen GH, Sampson L, Vivier PM, Galea S. Prevalence of Depression Symptoms in US Adults Before and During the COVID-19 Pandemic. JAMA Netw Open [Internet]. 2020 Sep 1 [cited 2021 Sep 11];3(9):e2019686–e2019686. Available from: https://jamanetwork.com/journals/jamanetworkopen/fullarticle/2770146 10.1001/jamanetworkopen.2020.19686 PMC7489837 32876685

[45] Akpalu J, Yorke E, Ainuson-Quampah J, Balogun W, Yeboah K. Depression and glycaemic control among type 2 diabetes patients: a cross-sectional study in a tertiary healthcare facility in Ghana. BMC Psychiatry 2018 18:1 [Internet]. 2018 Nov 6 [cited 2021 Sep 12];18(1):1–7. Available from: https://link.springer.com/articles/10.1186/s12888-018-1933-2 10.1186/s12888-018-1933-2 PMC6219193 30400843

[46] Brinda EM, Rajkumar AP, Attermann J, Gerdtham UG, Enemark U, Jacob KS. Health, Social, and Economic Variables Associated with Depression Among Older People in Low and Middle Income Countries: World Health Organization Study on Global AGEing and Adult Health. The American Journal of Geriatric Psychiatry. 2016 Dec 1;24(12):1196–208. 10.1016/j.jagp.2016.07.016 27743841

[47] Schuch FB, Vancampfort D, Firth J, Rosenbaum S, Ward PB, Silva ES et al. Physical Activity and Incident Depression: A Meta-Analysis of Prospective Cohort Studies. https://doi.org/101176/appi.ajp201817111194 [Internet]. 2018 Apr 25 [cited 2021 Sep 13];175(7):631–48. Available from: 10.1176/appi.ajp.2018.17111194 10.1176/appi.ajp.2018.17111194 29690792

[48] Marx W, Moseley G, Berk M, Jacka F. Nutritional psychiatry: the present state of the evidence. Proceedings of the Nutrition Society [Internet]. 2017 Nov 1 [cited 2021 Sep 13];76(4):427–36. Available from: https://www.cambridge.org/core/journals/proceedings-of-the-nutrition-society/article/nutritional-psychiatry-the-present-state-of-the-evidence/88924C819D21E3139FBC48D4D9DF0C08 10.1017/S0029665117002026 28942748

[49] Li Y, Lv MR, Wei YJ, Sun L, Zhang JX, Zhang HG, et al. Dietary patterns and depression risk: a meta-analysis. Psychiatry Res. 2017 Jul;1:253:373–82. 10.1016/j.psychres.2017.04.020 28431261

[50] Lassale C, Batty GD, Baghdadli A, Jacka F, Sánchez-Villegas A, Kivimäki M et al. Healthy dietary indices and risk of depressive outcomes: a systematic review and meta-analysis of observational studies. Molecular Psychiatry 2018 24:7 [Internet]. 2018 Sep 26 [cited 2021 Sep 13];24(7):965–86. Available from: https://www.nature.com/articles/s41380-018-0237-8 10.1038/s41380-018-0237-8 PMC6755986 30254236

[51] Anand A. Understanding Depression among Older Adults in Six Low-Middle Income Countries using WHO-SAGE Survey.Behavioral Health [Internet]. 2015 [cited 2021 Sep 13];1(2). Available from: www.jghcs.info

[52] Cochran WG. Sampling techniques. John Wiley & Sons; 2007.

[53] Zimmerman L, Olson H, Tsui A, Radloff S. Stud Fam Plann. 2017 Sep;48(1):293–303. PMA2020: Rapid Turn-Around Survey Data to Monitor Family Planning Service and Practice in Ten Countries. 10.1111/sifp.12031 PMC6084342 28885679

[54] Amissah J, Badu E, Agyei-Baffour P, Nakua EK, Mensah I. Predisposing factors influencing occupational injury among frontline building construction workers in Ghana. BMC Res Notes [Internet]. 2019 Dec 6;12(1):728. Available from: https://bmcresnotes.biomedcentral.com/articles/10.1186/s13104-019-4744-8 10.1186/s13104-019-4744-8 PMC6836387 31694711

[55] Amissah J, Agyei-Baffour P, Badu E, Agyeman JK, Badu ED. The Cost of Managing Occupational Injuries Among Frontline Construction Workers in Ghana.Value Health Reg Issues. 2019;19. 10.1016/j.vhri.2019.06.002 31377654

[56] Stone L, Heward J, Paddick SM, Dotchin CL, Walker RW, Collingwood C et al. Screening for Instrumental Activities of Daily Living in Sub-Saharan Africa: A Balance Between Task Shifting, Simplicity, Brevity, and Training. J Geriatr Psychiatry Neurol. 2018 Sep 1;31(5):248–55. 10.1177/0891988718790400 30049234

[57] Yesavage JA, Brink TL, Rose TL, Lum O, Huang V, Adey M et al. Development and validation of a geriatric depression screening scale: A preliminary report.J Psychiatr Res. 1982 Jan1;17(1):37–49. 10.1016/0022-3956(82)90033-4 7183759

[58] Sharp LK, Lipsky MS. Screening for Depression across the Lifespan: a review of measures for Use in Primary Care settings. Am Fam Physician. 2002 Sep;15(6):1001. 12358212

[59] García-Peña C, Wagner FA, Sánchez-Garcia S, Juárez-Cedillo T, Espinel-Bermúdez C, García-Gonzalez JJ et al. Depressive Symptoms Among Older Adults in Mexico City. Journal of General Internal Medicine 2008 23:12 [Internet]. 2008 Sep 26 [cited 2021 Sep 15];23(12):1973–80. Available from: https://link.springer.com/article/10.1007/s11606-008-0799-2 10.1007/s11606-008-0799-2 PMC2596501 18818976

[60] Boult C, Boult LB, Morishita L, Dowd B, Kane RL, Urdangarin CF. A Randomized Clinical Trial of Outpatient Geriatric Evaluation and Management. J Am Geriatr Soc [Internet]. 2001 Apr 1 [cited 2021 Sep 15];49(4):351–9. Available from: https://onlinelibrary.wiley.com/doi/full/10.1046/j.1532-5415.2001.49076.x 10.1046/j.1532-5415.2001.49076.x 11347776

[61] Salamero M, Marcos T. Factor study of the Geriatric Depression Scale. Acta Psychiatr Scand [Internet]. 1992 Oct 1 [cited 2021 Sep 15];86(4):283–6. Available from: https://onlinelibrary.wiley.com/doi/full/10.1111/j.1600-0447.1992.tb03267.x 10.1111/j.1600-0447.1992.tb03267.x 1456071

[62] Nyunt MSZ, Fones C, Niti M, Ng TP. Criterion-based validity and reliability of the Geriatric Depression Screening Scale (GDS-15) in a large validation sample of community-living Asian older adults. http://dx.doi.org/101080/13607860902861027 [Internet]. 2009 May [cited 2021 Sep 15];13(3):376–82. Available from: https://www.tandfonline.com/doi/abs/10.1080/13607860902861027 10.1080/13607860902861027 19484601

[63] Marfo CO, Donkor P, Twi Medical G. 2017 [cited 2023 Jan 2];(January). Available from: https://www.researchgate.net/publication/313114052

[64] Patel V, Abas M, Broadhead J, Todd C, Reeler A. Depression in developing countries: lessons from Zimbabwe. BMJ [Internet]. 2001 Feb 24 [cited 2021 Sep 9];322(7284):482–4. Available from: https://www.bmj.com/content/322/7284/482 10.1136/bmj.322.7284.482 PMC1119689 11222428

[65] Organization WH. Depression and other common mental disorders. global health estimates [Internet]. 2017 [cited 2021 Sep 9];48(1):56–60. Available from: https://apps.who.int/iris/handle/10665/254610

